# Tepotinib Inhibits the Epithelial–Mesenchymal Transition and Tumor Growth of Gastric Cancers by Increasing GSK3β, E-Cadherin, and Mucin 5AC and 6 Levels

**DOI:** 10.3390/ijms21176027

**Published:** 2020-08-21

**Authors:** Sung-Hwa Sohn, Hee Jung Sul, Bohyun Kim, Bum Jun Kim, Hyeong Su Kim, Dae Young Zang

**Affiliations:** 1 Hallym Translational Research Institute, Hallym University Sacred Heart Hospital, Anyang 14066, Korea; iisupy@korea.ac.kr (S.-H.S.); glwjd82@naver.com (H.J.S.); cowo7@naver.com (B.K.); 2 Department of Internal Medicine, Hallym University Medical Center, Hallym University College of Medicine, Anyang-si, Gyeonggi-do 14068, Korea; getwisdom1025@gmail.com (B.J.K.); nep2n74@gmail.com (H.S.K.)

**Keywords:** c-MET, EMT, gastric cancer, MUC5AC, MUC5B, MUC6, tepotinib

## Abstract

Aberrant expression of mucins (MUCs) can promote the epithelial–mesenchymal transition (EMT), which leads to enhanced tumorigenesis. Carcinogenesis-related pathways involving c-MET and β-catenin are associated with MUCs. In this study, we characterized the expression of EMT-relevant proteins including MET, β-catenin, and E-cadherin in human gastric cancer (GC) cell lines, and further characterized the differential susceptibility of these cell lines compared with the c-MET inhibitor tepotinib. We assessed the antitumor activity of tepotinib in GC cell lines. The effects of tepotinib on cell viability, apoptotic cell death, EMT, and c-MET and β-catenin signaling were evaluated by 3-(4,5 dimethylthiazol-2-yl)-5-(3-carboxymethoxyphenyl-2-(4-sulfophenyl)-2H-tetrazolium (MTS), flow cytometry, Western blotting, and qRT-PCR. The antitumor efficacy was assessed in MKN45 xenograft mice. Tepotinib treatment induced apoptosis in c-MET-amplified SNU620, MKN45, and KATO III cells, but had no effect on c-MET-reduced MKN28 or AGS cells. Tepotinib treatment also significantly reduced the protein levels of phosphorylated and total c-MET, phosphorylated and total ERK, β-catenin, and c-MYC in SNU620 and MKN45 cells. In contrast, this drug was only slightly active against KATO III cells. Notably, tepotinib significantly reduced the expression of EMT-promoting genes such as MMP7, COX-2, WNT1, MUC5B, and c-MYC in c-MET-amplified GC cells and increased the expression of EMT-suppressing genes such as MUC5AC, MUC6, GSK3β, and E-cadherin. In a mouse model, tepotinib exhibited good antitumor growth activity along with increased E-cadherin and decreased phosphorylated c-MET (phospho-c-MET) protein levels. Collectively, these results suggest that tepotinib suppresses tumor growth and migration by negatively regulating c-MET-induced EMT. These findings provide new insights into the mechanism by which MUC5AC and MUC6 contribute to GC progression.

## 1. Introduction

Mucins (MUCs) are part of carcinogenesis-related pathways, involving β-catenin, HGF/c-MET, NF-κB, MAPK, Ras/Erk, VEGF, c-JNK, and TGF-β [1,2,3,4]. MUCs are responsible for the formation of a protective mucous membrane barrier [5,6]. Aberrant expression of MUCs can promote the epithelial–mesenchymal transition (EMT) [7,8]. Among these MUCs, MUC5AC and MUC6 are specific to stomach mucus [9]. The stomach has a two-layered mucus system, consisting of an inner, attached mucus and an outer, unattached mucus layer, both comprised of MUC5AC, which acts as a diffusion barrier for hydrochloric acid [10,11,12,13] produced in stomach glands. These glands also secrete gel-forming MUC6 and pepsin [14].

The Wnt/β-catenin, HGF/c-MET, and Ras/Erk pathways have been associated with numerous cancers [15,16,17] and mediate EMT in gastric cancer (GC) [18,19,20]. The EMT process includes adherence junction components (e.g., E-cadherin (ECAD)), matrix metalloproteinase (MMP) 7 and 9, tumor microenvironment components (e.g., cyclooxygenase-2 (COX-2)), and c-MET [21,22,23]. Aberrantly activated c-MET can drive tumorigenesis, leading to aggressive cancer phenotypes and poor prognosis by promoting tumor cell survival, migration, EMT, and treatment resistance [23]. Therefore, c-MET has been highlighted as a promising target in GC. In particular, identifying proper GC subgroups is important for the selection of c-MET inhibitors. There is a need to define the c-MET levels using various GC cells with different c-MET expression statuses. The inhibitory activities of 1449 FDA-approved drugs were evaluated in SNU620 cells during the screening of new therapeutic agents for the treatment of c-MET-related GC. Among these drugs, tepotinib showed the highest inhibitory activity; therefore, this drug requires further examination. In the present study, we evaluated the effects of tepotinib on the suppression of GC proliferation, apoptosis, EMT, and tumor progression.

## 2. Results

### 2.1. Effective Dose of Tepotinib in c-MET-Positive Cells

**Figure 1:**
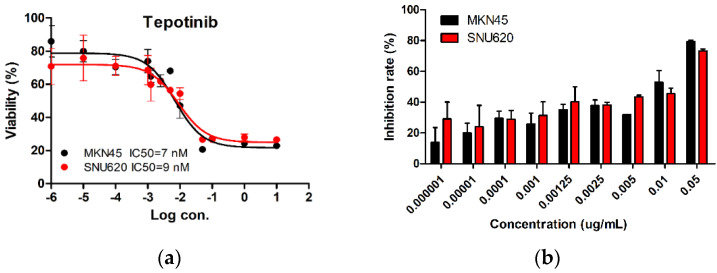
The effect of tepotinib in c-MET-amplified gastric cancer (GC) cells. SNU620 and MKN45 cells were treated with various concentrations of tepotinib for 48 h. (**a**) SNU620 and MKN45 cells were treated with tepotinib and viability was measured after 48 h. The IC_50_ for tepotinib and condition is shown in the figure. (**b**) Relationship between activity and concentration of tepotinib against SNU620 and MKN45 cells. Data are means ± standard deviation. IC_50_: average 50% inhibitory concentration.

We tested the dose-dependent inhibitory effects of tepotinib in c-MET-amplified SNU620 and MKN45 cells (Figure 1). The cells were treated with different concentrations of tepotinib for 48 h, and the optimal dose was determined by evaluating cell viability using an MTS assay. Tepotinib inhibited cell viability, with an average 50% inhibitory concentration (IC50) of 7 nM in MKN45 cells and 9 nM in SNU620 cells. It can be seen that the inhibition rate of tepotinib against SNU620 and MKN45 cell lines increased with increasing concentration. Tepotinib inhibited the growth of the SNU620 and MKN45 cells in a concentration-dependent manner.

### 2.2. Decreased Migration of c-MET-Amplified GC Cells by Tepotinib

**Figure 2:**
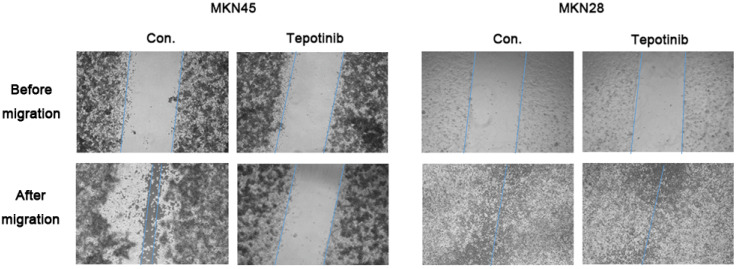
A wound-healing assay was used to assess the effect of tepotinib on the migration abilities of MKN28 and MKN45 cells. Tepotinib-treated MKN45 cells showed suppressed migration ability compared with tepotinib-treated MKN28 cells. Con, non-treated control cell lines.

c-MET induces cancer cell migration via the stimulation of cancer cell motility [23]. The migration rate of c-MET-amplified MKN45 cells was significantly decreased by tepotinib treatment compared with that of MKN28 cells (Figure 2). These findings indicate that tepotinib decreases the motility of c-MET-amplified GC cells.

### 2.3. Effect of Tepotinib on Cell Apoptosis

**Figure 3:**
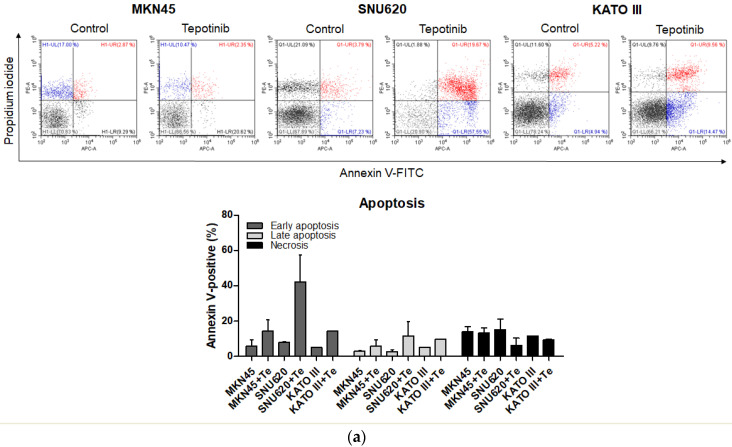

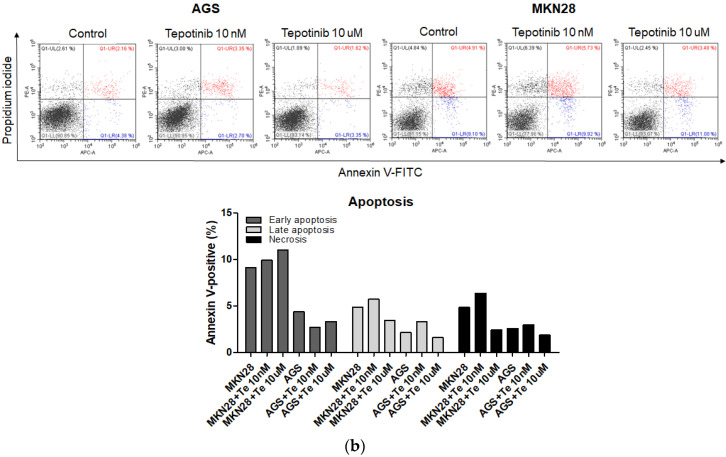
Apoptotic activation of c-MET-positive (**a**): MKN45, SNU620, and KATO III and c-MET-negative (**b**): AGS and MKN28 GC cells induced by tepotinib. Flow cytometric assay of the apoptotic and necrotic cells (UL: necrotic; UR: late apoptotic; LL: live; LR: early apoptotic) after 48 h of incubation with tepotinib (10 nM).

**Figure 4:**
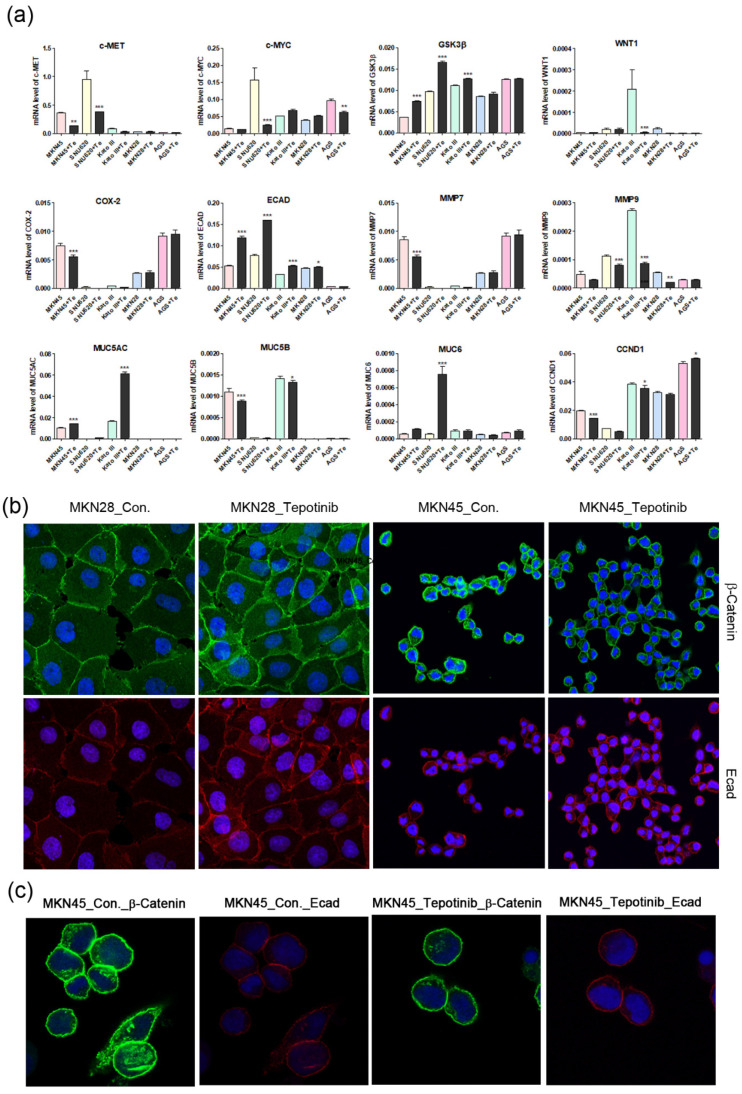
The effects of tepotinib on epithelial–mesenchymal transition genes and proteins in GC cells. (**a**) mRNA levels of WNT1, c-MYC, GSK3β, MMP7, COX-2, ECAD, MUC5AC, MUC5B, and MUC6 in MKN45, SNU620, MKN28, KATO III, and AGS cells were determined by quantitative reverse-transcription polymerase chain reaction after treatment with tepotinib (10 nM) for 48 h. Data are means ± standard deviation. Significant differences were evaluated by one-way ANOVA; * *P* < 0.05; ** *P* < 0.01; *** *P* < 0.001. (**b**) Immunofluorescence staining of ECAD and β-catenin using 4′,6-diamidino-2-phenylindole in MKN28 and MKN45 cells treated with tepotinib (magnification ×400). (**c**) Immunofluorescence staining of ECAD and β-catenin in MKN45 cells treated with tepotinib (magnification ×1000).

To evaluate whether tepotinib induces cell death differentially depending on c-MET status, we used various GC cells with different c-MET expression statuses (MKN45 and SNU620 cells: high-c-MET; KATO III cells: low-c-MET; MKN28 and AGS cells: very low-c-MET) to assess early apoptotic, late apoptotic, and necrotic cell populations (Figure 3 and Figure 4a). Tepotinib significantly induced apoptosis in SNU620, MKN45, and KATO III cells, whereas apoptosis was rarely observed in MKN28 and AGS cells (Figure 3). The percentages of early and late apoptotic cells were 53.88% (vs. 10.48% in the control), 20.11% (vs. 8.7% in the control), and 23.96% (vs. 10.16% in the control) after exposure to tepotinib for 48 h in SNU620, MKN45, and KATO III cells, respectively (Figure 3a). MKN45 and KATO III cell lines have high MUC5B expression (Figure 4a), and MUC5B plays a role in cancer cell resistance to chemotherapeutic drugs [24]. Nevertheless, these data suggested that tepotinib promoted apoptosis in c-MET-positive GC cells.

### 2.4. Tepotinib Inhibition of c-MET Activation and EMT in c-MET-Amplified GC Cells

**Figure 5:**
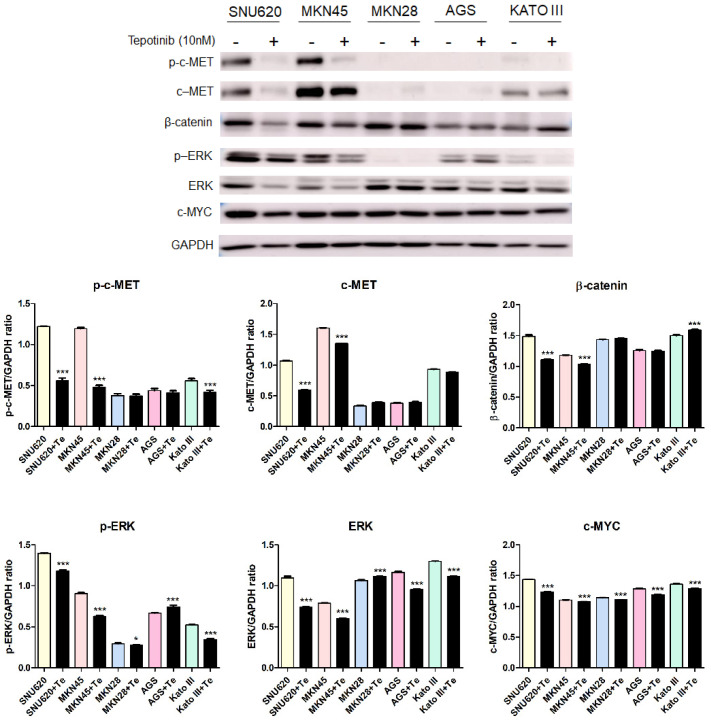
The effect of tepotinib on c-MET, β-catenin, ERK, and c-MYC protein levels in GC cells. The protein levels of phosphorylated and total c-MET, β-catenin, phosphorylated and total ERK, and c-MYC in MKN45, SNU620, MKN28, KATO III, and AGS cells were determined by Western blot analyses after treatment with tepotinib (10 nM) for 48 h. Data represent the means ± SD. Significance was evaluated by one-way ANOVA; * *P* < 0.05; *** *P* < 0.001.

To further investigate the mechanisms of tepotinib in GC cells with different c-MET expression statuses (high-c-MET, low-c-MET, and very low-c-MET), the gene and protein expression of EMT marker was analyzed. Tepotinib treatment decreased EMT-promoting genes such as MUC5B, MMP7, MMP9, COX-2, WNT1, CCND1, and c-MYC and increased EMT-suppressing genes such as MUC5AC, MUC6, GSK3β, and ECAD in c-MET-amplified GC cells (Figure 4a). Analysis of EMT marker protein expression by immunofluorescence revealed that tepotinib suppressed EMT compared with control cells (Figure 4). The ECAD protein level was slightly increased in c-MET-amplified MKN45 cells, whereas that of β-catenin was markedly downregulated compared with control cells (Figure 4c). However, ECAD and β-catenin protein levels were increased in c-MET-negative MKN28 cells. Tepotinib also significantly suppressed the protein levels of phosphorylated and total c-MET, phosphorylated and total ERK, β-catenin, and c-MYC in SNU620 and MKN45 cells. These drugs were only minimally active against KATO III cells, whereas the effects of tepotinib were seldom observed in MKN28 and AGS cells (Figure 5).

### 2.5. Tepotinib Inhibits the Growth of Subcutaneous Xenograft Tumors in Nude Mice

**Figure 6:**
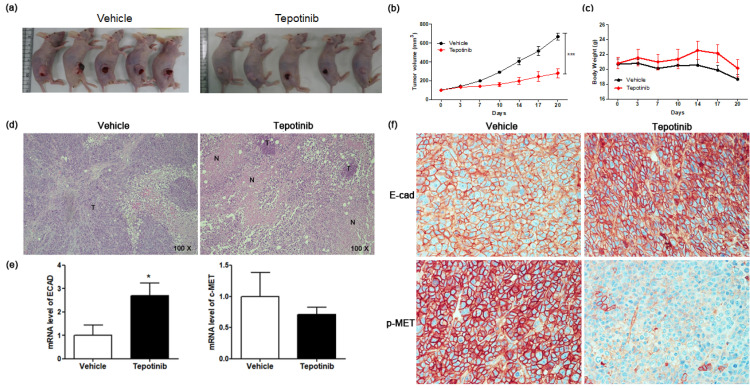
The effect of tepotinib on MKN45 xenograft tumor growth in nude mice. (**a**) Reduced xenograft tumor size by tepotinib treatment. (**b**) Volume (mm^3^) of the MKN45 xenograft tumors, measured twice per week. Significance was evaluated by two-way ANOVA; *** *P* < 0.001 compared with the vehicle-treated control. (**c**) Change in body weight (**g**) in a xenograft mouse. (**d**) Histological analysis of xenograft tumors in the presence versus absence of tepotinib treatment (magnification ×100). Untreated control xenografts had differentiated carcinoma cells without necrosis. A tepotinib-treated xenograft tumor showed increased scattered necrotic lesions. (**e**) mRNA levels of ECAD and c-MET in a xenograft mouse were determined by quantitative reverse-transcription polymerase chain reaction. Data are means ± standard deviation. Significant differences were evaluated by t-test; * *P* < 0.05. (**f**) Immunohistochemical analysis of ECAD and p-MET in the MKN45 tumor sections treatment (magnification ×400).

To test the efficacy of tepotinib on GC cells expressing high levels of MUC5B and c-MET in vivo, we subcutaneously implanted MKN45 cells into the right flanks of nude mice (Figure 6a). Mice were administered tepotinib (10 mg/kg body weight) or vehicle by oral gavage daily. We measured tumor volume twice weekly to monitor growth (Figure 6b). Tumor volume was significantly inhibited in the tepotinib-treated group compared with the control group (vehicle). At the end of the experiment, there was no significant difference in body weight between the groups (Figure 6c). Necrosis is often induced in tumors after treatment with anticancer agents; indeed, hematoxylin and eosin staining revealed more necrosis in the tepotinib-treated group than in the control group (Figure 6d). Gene expression analyses of ECAD and c-MET were performed in xenograft sections (Figure 6e). The tepotinib-treated group had significantly higher ECAD levels but lower c-MET levels compared with the vehicle control group, indicating that tepotinib exhibited the suppression of invasive growth with increased ECAD and decreased c-MET gene levels. Immunohistochemical analyses of ECAD and p-MET were performed in xenograft sections (Figure 6f). The tepotinib-treated group had significantly higher ECAD levels but significantly lower p-MET levels compared with the vehicle control group, indicating that tepotinib exhibited good antitumor growth activity with increased ECAD and decreased p-MET protein levels.

## 3. Discussion

Rates of GC incidence are markedly increased in Eastern Asia compared with the rest of the world [25]; the highest rates for both sexes worldwide occur in the Republic of Korea. Complete resection of localized tumors is the most common treatment for GC; however, patients are usually diagnosed at an advanced stage [26]. Despite improvements in surgical treatment and chemotherapy, patients with unresectable tumors have a median survival of only 10 months [27]. Improving the prognoses of advanced GC patients requires treatments involving chemotherapy and surgery. Carcinogenesis pathways consisting of c-MET and β-catenin involve MUCs, which are frequently dysregulated and strongly implicated in GC progression, including EMT [4,17,28]. GC progression involves proliferation and metastasis via EMT, which promotes invasion, adhesion, extravasation, and regrowth in different organs [21,29,30]. MUC1 and MUC4 overexpression induces the EMT in renal carcinoma and ovarian cancer via the Wnt/β-catenin pathway [31,32], whereas MUC16 knockdown induces EMT [33]. MUC5AC and MUC6 are the main MUC components of the stomach [9]. The mRNA levels of MUC5B are increased in gastrointestinal cancers, but those of MUC5AC and MUC6 are decreased in GC [34]. MUC5B affects gastrointestinal carcinogenesis by inducing the Wnt/β-catenin pathway [35]. In addition, MUC5B expression has been positively correlated with the poor prognoses of gastric carcinomas [36,37,38]. In the present study, we investigated the effects of inhibiting MUC5B, c-MET, and β-catenin signaling by treatment with tepotinib, which promotes EMT inhibitory genes such as MUC5AC, MUC6, GSK3β, and ECAD. Tepotinib showed the greatest inhibition of these genes, but a low rate of apoptosis in MKN45 and KATO III cells compared with SNU620 cells. MKN45 and KATO III are high-MUC5B-expressing GC cell lines, and MUC5B-positive GC cells show increased resistance to chemotherapy-induced cell death [24]. EMT results in a decrease in ECAD expression and an increase in c-MET and MMP expression [38]. COX-2 has been shown to activate cytoplasmic β-catenin, inducing the activation of MMPs [39,40]. MMPs cleave ECAD, leading to the relocalization of β-catenin to the nucleus and induction of EMT [41]. Tepotinib treatment of c-MET-positive GC cells suppressed EMT via increased expression of GSK3β, ECAD, MUC5AC, and MUC6 and decreased expression of MMP7, COX-2, and MUC5B. Our results suggest that tepotinib inhibits Wnt/β-catenin signaling and the c-MET and ERK pathways by inhibiting c-MET and ERK phosphorylation and c-MYC and β-catenin expression in c-MET-positive GC cells. To further examine the antitumor effect of tepotinib in high-MUC5B- and -c-MET-expressing GCs, we conducted an in vivo study using xenograft mice and found that tepotinib significantly inhibited tumor growth.

## 4. Materials and Methods

### 4.1. Cell Culture and Reagents

The SNU620, MKN45, MKN28, KATO III, and AGS gastric cell lines were obtained from the Korean Cell Line Bank (Seoul, Korea) and maintained in RPMI 1640 supplemented with 10% fetal bovine serum. The cells were cultured in 100% humidity and 5% CO_2_ at 37 °C. The c-MET inhibitor drug (tepotinib) was purchased from Selleck Chemicals (Houston, TX, USA). The annexin V-APC/propidium iodide (PI) apoptosis detection kit (Thermo Fisher Scientific, Rockford, IL, USA) was used in the present study.

### 4.2. Growth Inhibition Assays

The IC50 of tepotinib in MKN45 and SNU620 cells was measured using the MTS assay for tepotinib at concentrations of 10, 1, 0.1, 0.05, 0.0025, 0.00125, 0.001, 0.0001, 0.00001, or 0.000001 µM for 48 h. On the day of the proliferation assay, the medium was removed, and 200 µL of fresh medium was added to each well of the 96-well plate, followed by 20 µL of MTS solution, and the plates were incubated at 37 °C for 1 h in a humidified environment with 5% CO_2_. The absorbance was measured at 490 nm using a microplate reader (Synergy 2 Multi-Mode Microplate Reader; BioTek, Winooski, VT, USA). The IC_50_ was calculated by nonlinear regression analysis using Prism software (GraphPad Software, San Diego, CA, USA).

### 4.3. Cell Migration Analysis

Migration assays were performed as described previously [42]. When cells reached confluence, a p200 pipette tip was used to scrape a straight line through the cell monolayer. The cells were then washed with phosphate-buffered saline (PBS) and further cultured with or without tepotinib in RPMI 1640. After incubation for 72 h, the gap width of the scratch was photographed and compared with the initial gap size at 0 h.

### 4.4. Apoptosis Analysis

The MKN45, SNU620, MKN28, KATO III, and AGS cells were seeded into 6-well plates at a density of 5 × 10^4^ cells/mL and were then treated with 10 nM or 10 µM of tepotinib. Cell death was determined using the annexin V-APC/PI apoptosis detection kit (Thermo Fisher Scientific, Waltham, MA, USA) using a CytoFLEX flow cytometer (Beckman Coulter, Brea, CA, USA). The percentages of intact and apoptotic cells were calculated using CytExpert software (Beckman Coulter).

### 4.5. Quantitative Real-Time PCR (qRT-PCR) Analysis

To quantitate mRNA expression, total RNA from each sample was reverse-transcribed into cDNA using the High-Capacity cDNA Reverse Transcription Kit (Applied Biosystems, Foster City, CA, USA). The qRT-PCR was performed using the Power SYBR Green PCR Master Mix and a LightCycler 96 instrument (Roche Applied Science, Indianapolis, IN, USA). The transcript levels of glyceraldehyde 3-phosphate dehydrogenase (GAPDH) were used for sample normalization. The primer sequences used were as follows: WNT1 (forward: 5′-TCC TCC ACG AAC CTG CTT AC-3′, reverse: 5′-CGG ATT TTG GCG TAT CAG AC-3′); MMP7 (forward: 5′-GGA GCT CAT GGG GAC TCC TA-3′, reverse: 5′-CCA GCG TTC ATC CTC ATC GA-3′); GSK3β (forward: 5′-GAA CTC CAA CAA GGG AGC AA-3′, reverse: 5′-GGG TCG GAA GAC CTT AGT CC-3′); MUC5AC (forward: 5′CCT TCG ACG GAC AGA GCT AC-3′, reverse: 5′-TCT CGG TGA CAA CAC GAA AG-3′); MUC5B (forward: 5′-TCC ACT ATG AGT GCG AGT GC-3′, reverse: 5′-AAG CGT GCA TGG ATC TCT CT-3′); MUC6 (forward: 5′-GCC TGC AAC TAC GAG GAG AC-3′, reverse: 5′-GAT GGT GCA GTT GTC CAC AC-3′); ECAD (forward: 5′-TGG GCC AGG AAA TCA CAT CC-3′, reverse: 5′-GGC ACC AGT GTC CGG ATT AA-3′); COX-2 (forward: 5′-TGA GCA TCT ACG GTT TGC TG-3′, reverse: 5′-AAC TGC TCA TCA CCC CAT TC-3′); c-MYC (forward: 5′-TCA AGA GGC GAA CAC ACA AC-3′, reverse: 5′-GGC CTT TTC ATT GTT TTC CA-3′); and glyceraldehyde 3-phosphate dehydrogenase (GAPDH) (forward: 5′-TTC ACC ACC ATG GAG AAG GC-3′, reverse: 5′-GGC ATG GAC TGT GGT CAT GA-3′).

### 4.6. Immunofluorescence Microscopy

MKN28 and MKN45 cells cultured on chamber slides were washed with PBS, fixed with 4% paraformaldehyde, and then incubated with an anti-ECAD (R & D systems, Abingdon, UK; 1:200 dilution) or anti-β-catenin (BD Transduction Laboratories, San Jose, CA, USA; 1:200 dilution) monoclonal antibody and then with an anti-mouse IgG Alexa Fluor 488 antibody (Invitrogen, Carlsbad, CA, USA) or an anti-rabbit IgG Alexa Fluor 568 antibody (Invitrogen, Carlsbad, CA, USA). Cells were examined using a confocal laser scanning microscope (LSM700; Carl Zeiss, Oberkochen, Germany).

### 4.7. Western Analysis

Western blot analysis was conducted using standard procedures. The commercially available primary antibodies were directed against anti-phospho-c-MET (Tyr1234/1235; 1:1000; #3077; Cell Signaling Technology, Danvers, MA, USA), anti-c-MET (1:1000; #4560; Cell Signaling Technology), anti-phospho-ERK (1:1000; #9101; Cell Signaling Technology), anti-ERK (1:1000; sc514302; Santa Cruz Biotechnology, Santa Cruz, CA, USA), anti-β-catenin (1:1000; #610153; BD Biosciences, San Jose, CA, USA), anti-c-MYC (1:1000; sc40; Santa Cruz Biotechnology), and anti-GAPDH (1:4000; sc32233; Santa Cruz Biotechnology).

### 4.8. Mice Xenograft Study

All experiments and animal handling procedures were approved by the Animal Experimental Ethics Committee of the Asan Medical Center, Seoul, Republic of Korea (IACUC No. 2018-12-299). All protocols were performed in accordance with the relevant guidelines and regulations. Six-week-old male BALB/c-nu/nu mice (Joongang Laboratory Animals, Seoul, Korea) were housed in cages, and maintained at 23 °C with a 12 h light/dark cycle under specific pathogen-free conditions. Each mouse was inoculated subcutaneously into the right flank with 1 × 10^7^ cells/mouse of the MKN45 human GC cell line. When the average tumor volume reached 100 mm^3^, the mice were randomly divided into various treatment and control groups (five mice per group). Tepotinib was formulated at 10 μg/g bodyweight in corn oil with 5% dimethyl sulfoxide. Tepotinib was administered daily for 3 weeks by oral gavage. The maximum volume per mouse was 100 μL. The tumor size was measured twice every week with a caliper (calculated volume = shortest diameter^2^ × longest diameter/2). The body weight and tumor size were recorded twice every week. After 3 weeks, the mice were euthanized and solid tumors were removed for further analysis.

### 4.9. Histology and Immunohistochemistry

MKN45 tumor tissues were fixed in 4% paraformaldehyde and then embedded in paraffin for hematoxylin and eosin staining and immunostaining. Tumor tissue sections (4 μm thick) were treated with 0.3% H_2_O/methanol for 20 min to block endogenous peroxidase and then incubated at 4 °C overnight with an anti-ECAD primary antibody (1:100 dilution) or anti-p-MET primary antibody (1:100). The slides were incubated with an avidin–biotin peroxidase complex (ABC kit, Vector Laboratories, Burlingame, CA, USA), and then the color was developed using 3,3′-diaminobenzidine tetrachloride (Zymed Laboratories, San Francisco, CA, USA). After immunohistochemical staining, the slides were counterstained with Harris’s hematoxylin for 1 min and then mounted with Canada balsam (Show Chemical Co. Ltd., Tokyo, Japan). Histology was evaluated by a pathologist in a blinded manner.

### 4.10. Statistical Analysis

The data were statistically analyzed using Prism 5 software (GraphPad Software). All values are presented as the mean ± standard deviation. Statistical significance was determined using one-way ANOVA. A *P* value < 0.05 indicated statistical significance.

## 5. Conclusions

The results of this study indicated that c-MET and MUCs were differentially expressed in GCs, and that tepotinib showed a significant inhibitory activity in c-MET- and MUC5B-expressed GCs. Our in vitro and in vivo studies strongly supported the clinical evaluations of tepotinib, which prevented the EMT and tumor growth in c-MET- and MUC5B-expressing GCs.

## Abbreviations

EMT	Epithelial–mesenchymal transition
GC	Gastric cancer
MUCs	Mucins
ECAD	E-cadherin
MMP7	Matrix metalloproteinase 7
COX-2	Cyclooxygenase-2
